# A Critical Case of Streptococcal Toxic Shock Syndrome: A Case Report

**DOI:** 10.7759/cureus.56170

**Published:** 2024-03-14

**Authors:** Lisandra Nunez Cuello, Deeksha Bhattarai, Yong Shin

**Affiliations:** 1 Internal Medicine, Danbury Hospital, Danbury, USA

**Keywords:** community acquired pneumonia, hypoxic respiratory failure, multiple organ failure, streptococcal toxic shock syndrome (stss), streptococcus pyogenes infection

## Abstract

A 41-year-old woman with a history of asthma presented to the emergency department with complaints of progressive malaise, dyspnea, vomiting, and diarrhea for a week. Upon presentation, the patient was hemodynamically unstable and exhibited severe respiratory distress. A chest computed tomography revealed consolidation of the left upper lobe with confluence in the left perihilar region and a left pleural effusion. The patient was admitted to the intensive care unit for further management of respiratory failure, and a chest tube was placed on the left side. Despite the absence of bacteremia, the diagnosis of Streptococcal toxic shock syndrome was confirmed through a pleural fluid culture positive for Streptococcus pyogenes and evidence of multiorgan failure. Her treatment included vasopressors, broad-spectrum antibiotics, and intravenous immunoglobulin. For renal failure, the patient required continuous renal replacement therapy. Despite all these interventions, the patient continued to decline, and left-sided video-assisted thoracoscopic surgery was pursued with subsequent improvement of her condition. The incidence of invasive Group A streptococcal disease is significant, with notable mortality and morbidity among affected patients. The rapid deterioration is thought to be secondary to the highly virulent nature of the pathogen and the production of superantigens. The rapid institution of adequate antibiotic coverage with beta-lactams and clindamycin has been shown to decrease the mortality rate. Intravenous immunoglobulin has also been included with a promising positive effect.

## Introduction

Streptococcal toxic shock syndrome (STSS) is an immune-mediated systemic inflammatory response to an infection in which exotoxins act as superantigens, causing massive cytokine release and increasing capillary permeability, ultimately leading to shock and multi-organ dysfunction. This is commonly seen with soft tissue infections, necrotizing fasciitis, myonecrosis, and bacteremia caused by toxic strains of group A streptococcus [1].

In 2021, the Centers for Disease Control and Prevention (CDC) estimated that the national incidence rate for invasive group A streptococcal (GAS) disease was 6.30 cases per 100,000 individuals. In this case report, we discuss a patient who developed STSS after an upper respiratory infection (URI) that required admission to the intensive care unit (ICU). The clinical course deteriorated, necessitating urgent hemodialysis (HD) and continuous renal replacement therapy (CRRT). The pleural fluid culture and multiorgan failure confirmed the diagnosis of STSS. We aim to emphasize the importance of early intervention in managing STSS, particularly those presenting after URI.

## Case presentation

A 41-year-old woman with a past medical history of asthma presented to the emergency department with complaints of progressive malaise, dyspnea, vomiting, and diarrhea for a week. She had visited her pulmonologist and was prescribed prednisone, azithromycin, and a bronchodilator. However, she experienced worsening symptoms which prompted her to come to the hospital. Upon further investigation, her husband reported that several family members had been diagnosed with streptococcal pharyngitis.

**Figure 1:**
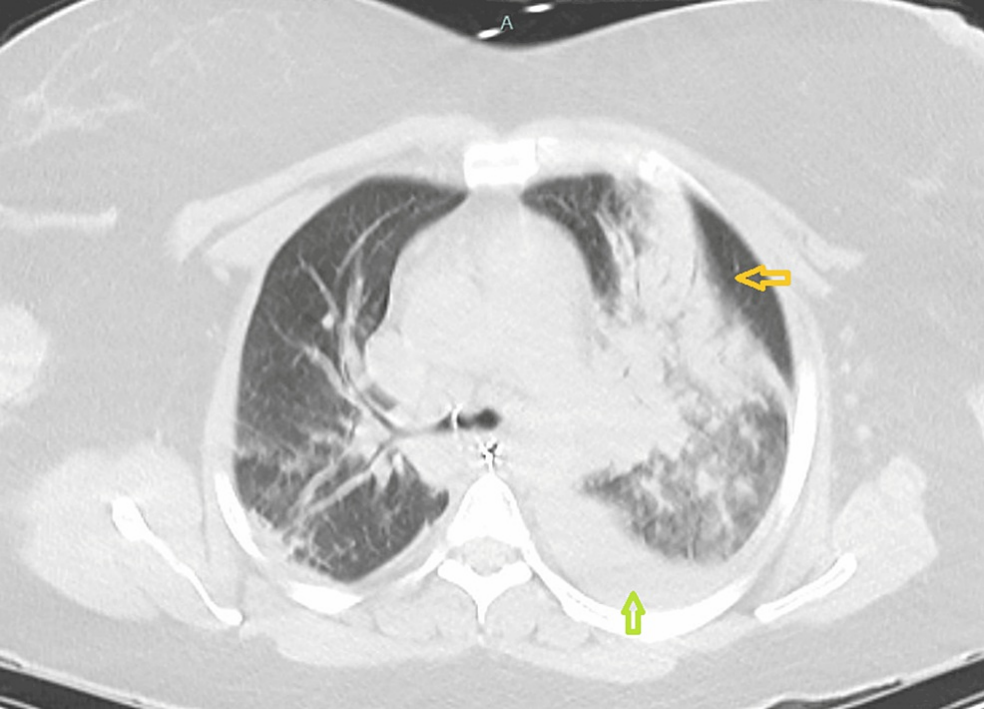
CT of the chest without contrast, axial cuts, showed a large consolidation of the left upper lobe with confluence in the left perihilar region (yellow arrow) and a left pleural effusion (green arrow).

On physical examination, she was afebrile, her pulse was 156 beats/minute, blood pressure was 93/67 mmHg, respiratory rate was 37 breaths/min, oxygen saturation was 92% on room air, she was extremely dyspneic and agitated, breath sounds were rhonchorous in both lung field. Her laboratory values are presented in Table 1. A computed tomography (CT) chest, abdomen, and pelvis without contrast was obtained, as shown in Figure 1, revealing consolidation of the left upper lobe with confluence in the left perihilar region and a left pleural effusion. The patient underwent emergent intubation due to increased work of breathing and hypoxia despite the use of noninvasive ventilation. ABG after intubation is listed in Table 2. In response to persistent hypotension, aggressive IV fluid resuscitation and vasopressors were initiated. For antibiotic coverage, she was started on vancomycin, cefepime, and clindamycin. A left-sided chest tube was placed with drainage of purulent fluid.

**Table 1:** Laboratory values. BUN: blood urea nitrogen; ALP: alkaline phosphatase; ALT: alanine aminotransferase; AST: aspartate aminotransferase; CK: creatine kinase; Pro-BNP: pro-brain natriuretic peptide; WBC: white blood cell.

Laboratory investigation	Day 1	Day 2	Day 3	Reference range
	Results			
Sodium	131 mmol/L	129 mmol/L	130 mmol/L	135-145
Potassium	3.6 mmol/L	3.9 mmol/L	4.2 mmol/L	3.5-5.3
Chloride	92 mmol/L	91 mmol/L	93 mmol/L	97-107
Bicarbonate	16 mmol/L	18 mmol/L	17 mmol/L	22-29
BUN	27 mg/dL	37 mg/dL	24 mg/dL	6-23
Creatinine	3.8 mg/dL	4.4 mg/dL	2.85 mg/dL	0.67-1.23
Lactic acid	11.4 mmol/L	7.3 mmol/L	9.3 mmol/L	<1.9
Phosphorus	7.1 mg/dL	4.7 mg/dL	5.2 mg/dL	2.6-4.7
Alkaline Phosphate	98 U/L	55 U/L	123 U/L	30-101
ALT	40 U/L	790 U/L	2725 U/L	10-55
AST	55 U/L	1578 U/L	3610 U/L	10-50
CK	2,901 U/L	7,137 U/L	12,772 U/L	26-192
Pro-BNP	27,015 pg/mL			<299
WBC	6.4 x10(9)/L	16.6 x10(9)/L	21.4 x10(9)/L	3.5-10
Hemoglobin	14.5 g/dL	10.9 g/dL	10.6 g/dL	13.5-17
Hematocrit	44.2 %	33.4 %	33.1 %	35.0-46.0
Platelets	281 x10(9)/L	194 x10(9)/L	109 x10(9)/L	150-400

**Table 2:** Arterial blood gas after intubation. pCO2: partial pressure of carbon dioxide, HCO3: bicarbonate, pO2: partial pressure of oxygen, O2 Saturation: oxygen saturation.

Arterial Blood Gas	Results	Reference range
pH	7.09	7.31-7.42
pCO2	62 mmHg	35-48
HCO3	19 mmol/L	22-26
pO2	153 mmHg	>70
O2 Arterial Saturation	98%	>95

**Figure 2:**
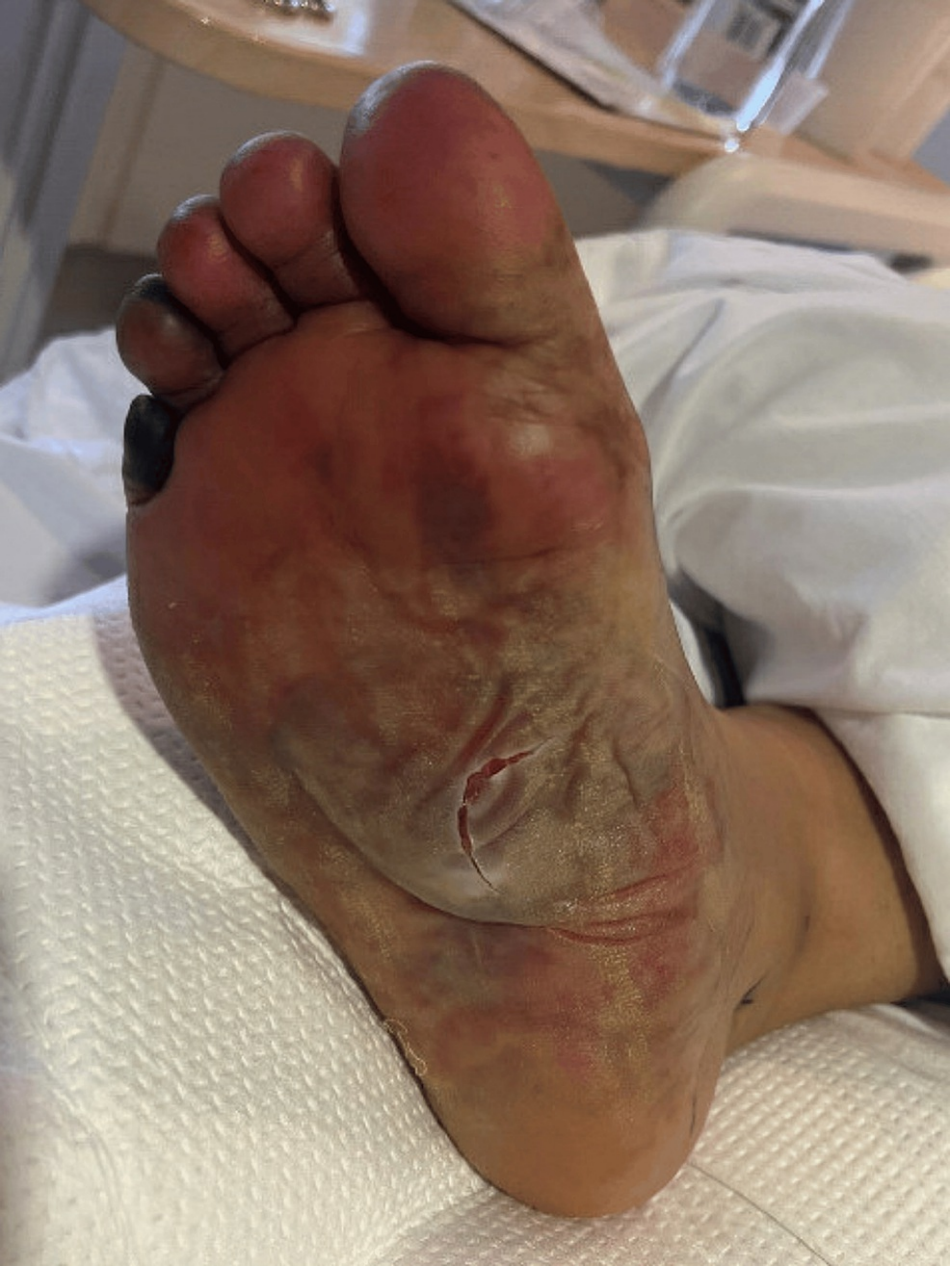
Desquamating blisters.

The patient was admitted to the ICU for further management of septic shock with multiorgan failure secondary to pneumonia with parapneumonic effusion. Unfortunately, her renal function continued to decline despite aggressive fluid resuscitation and maximal dosages of vasopressors (norepinephrine, phenylephrine, vasopressin, and epinephrine). On the third day of hospitalization, the patient became anuric, leading to consultation with Nephrology for continuous venovenous hemofiltration (CVVH). Meanwhile, the left pleural fluid and lower respiratory culture started growing Streptococcus pyogenes. Blood culture showed no growth, and tests for Legionella antigen and MRSA PCR were negative. Infectious disease consultation recommended de-escalation of antibiotics to cefazolin and clindamycin, and intravenous immunoglobulin (IVIG) was added. Her overall presentation was consistent with STSS, including renal failure, rhabdomyolysis, shock liver, coagulopathy, thrombocytopenia, progressive ARDS, and acute limb ischemia of bilateral upper extremities secondary to hypoperfusion from septic shock. Desquamating blisters and necrosis of the right fifth digit were observed in Figure 2, prompting consultation with a podiatrist, who recommended dressing changes, which led to an improvement in the skin changes.

Serial chest x-rays were obtained to monitor for recollection of empyema. The patient was gradually weaned off vasopressors, and CVVH contributed to the improvement of her volume status, thereby improving oxygenation. On the sixth day of the hospital stay, after successfully tolerating a trial of pressure-support ventilation (PSV), she was extubated, and the chest tube was removed. Subsequently, CVVH was transitioned to intermittent HD, and antibiotics were de-escalated to amoxicillin-clavulanate in preparation for discharge. However, the patient developed a fever, prompting a repeat CT chest, which revealed an increasing left-sided pleural effusion, concerning the recurrence of empyema. Thoracic surgery was consulted, and she underwent left-sided video-assisted thoracoscopic surgery (VATS) with evacuation of blood and fluid from the left chest and decortication of the left lung for source control. Her condition gradually improved, and she was discharged to an acute inpatient rehabilitation facility on room air and without requiring HD. She was prescribed amoxicillin-clavulanate to complete a six-week course. The patient has been following up with nephrology, infectious disease, and podiatry. Currently, she is undergoing evaluation for the possibility of toe(s) resection due to gangrenous changes resulting from limb ischemia during her hospital stay.

## Discussion

Invasive GAS infections have been described by the presence of GAS in normally sterile sites. These infections carry a high mortality and morbidity for the affected patients, especially those with Streptococcus pyogenes pneumonia evolving into STSS [3].

Given the severity of these cases, most patients will require ICU care. Those with Streptococcal pneumonia have a mortality rate ranging from 15% to 38%, and if it progresses to STSS, the mortality rate can further increase to 30%-70% [4,5]. The initial presentation of our patient aligns with existing literature, where individuals with pneumonia commonly manifest symptoms such as dyspnea, malaise, and cough following a viral prodrome. The rapid deterioration is thought to be secondary to the highly virulent nature and the production of superantigens, leading to multiorgan failure and STSS. Patients may exhibit symptoms such as fever or hypothermia, desquamating rash, and confusion. Nearly 50% of patients will have normal blood pressure on presentation; however, continuous monitoring and resuscitation should be provided, as they might develop hypotension in the next four to 24 hours [6,7].

The major virulence factor of GAS is the M protein, which enables the organism to invade sterile sites, evade phagocytosis, and colonize by attaching to epithelial cells. GAS is also capable of secreting an exotoxin that acts as a superantigen. The best-described superantigen is exotoxin A, which, through the stimulation of a large proportion of circulating T cells, creates a massive cytokine release leading to capillary leak and profound hypotension. This process initiates an inflammatory cascade responsible for STSS [8].

Due to the aggressive nature of the disease, the initial workup for these patients should include a “rainbow of laboratory tests.” Initially, as observed in our patient, white blood cell counts may be normal or only mildly elevated; however, particular attention should be given to immature circulating neutrophils. Hemolytic anemia should also be considered if hemoglobin levels start to drop, possibly due to the effects of hemolysins. Generally, patients will present with multi-organ failure, as indicated by laboratory results such as increased creatinine levels, low platelets, abnormal DIC panel, and normal or elevated liver enzymes [9]. It is important to perform serial laboratory assessments throughout the course of this disease, given its rapid evolution and severity.

The management of STSS requires a multidisciplinary approach, rapid source control, and early initiation of effective antibiotic therapy. Extensive supportive care, including aggressive fluid replacement and often the initiation of inotropes. Eventually, patients with STSS may develop significant hypoxia requiring endotracheal intubation and ventilatory support, as well as renal replacement [5].

The first antibiotic of choice, once the organism has been isolated from the sterile site (as in our patient's pleural fluid), will be beta-lactam antibiotics which are also effective against Streptococcus pyogenes. However, if there is a high suspicion of methicillin-resistant S. aureus (MRSA), vancomycin should be considered [3,10].

Clindamycin is crucial in the early treatment regimen for STSS due to its ability to neutralize the effects of superantigens. Furthermore, it can potentiate phagocytosis by inhibiting the M-protein and overcoming the so-called Eagle effect - a phenomenon where high bacterial concentrations might prevent bacterial replication, preventing antibiotics like beta-lactams from being effective and given the mechanism of action of clindamycin, it allows the antibiotic to combat the infection. Clindamycin is recommended not as a standalone agent but as an adjunct to beta-lactam antibiotics, particularly penicillin or cephalosporins, to maximize coverage [6].

Linezolid could also be considered instead of clindamycin due to concerns about rising clindamycin resistance, its broad coverage against MRSA, and the lower risk of Clostridium difficile infection compared to clindamycin. However, studies have yet to be performed to compare these antibiotics, and the potential mortality benefit should also be evaluated [11]. The administration of IVIG to clindamycin-treated patients with STSS has been shown to reduce mortality by neutralizing superantigens and increasing bacterial clearance. However, it is crucial to consider that these studies have always been performed with the use of clindamycin, which could potentially bias the results [12].

Given the severe nature of STSS, we must also consider the increased morbidity that accompanies this disease. The organ failure characteristic of the disease raises the risk of chronic respiratory failure, the need for renal replacement, or surgical debridement. Prolonged hospitalization will increase the risk of extended post-hospital rehabilitation. Therefore, it is recommended to ensure that these patients receive adequate nutrition, physical rehabilitation, and support systems for better outcomes [13].

## Conclusions

STSS is aggressive in nature, usually requiring intensive care because of the high morbidity and mortality rates. Management should involve a multidisciplinary approach, focusing on early, effective antibiotic coverage, source control with chest tube placement or VATS procedure, and early rehabilitation for patients who have experienced prolonged hospitalization.
